# Bis{(*E*)-2-[(2-chloro-3-pyrid­yl)imino­meth­yl]-6-meth­oxy­phenolato-κ^2^ *N*,*O* ^1^}copper(II)

**DOI:** 10.1107/S1600536810052414

**Published:** 2010-12-18

**Authors:** Wen-Kui Dong, Yuan Wang, Jian-Chao Wu, Shou-Ting Zhang

**Affiliations:** a School of Chemical and Biological Engineering, Lanzhou Jiaotong University, Lanzhou 730070, People’s Republic of China

## Abstract

In the title mononuclear copper(II) complex, [Cu(C_13_H_10_ClN_2_O_2_)_2_], the Cu^II^ ion, lying on an inversion center, is four-coordinated in a *trans*-CuN_2_O_2_ square-planar geometry by two phenolate O and two imino N atoms from two symmetry-related *N*,*O*-bidentate Schiff base ligands. The shortest Cu⋯Cu distance is 7.5743 (9) Å. However, there are weak intra­molecular electrostatic inter­actions between the Cu atom and the Cl atom of the ligand, with a Cu⋯Cl distance of 3.3845 (9) Å.

## Related literature

For the synthesis and related crystal strcutures, see: Dong *et al.* (2009 ▶, 2010 ▶); Ding *et al.* (2009 ▶).
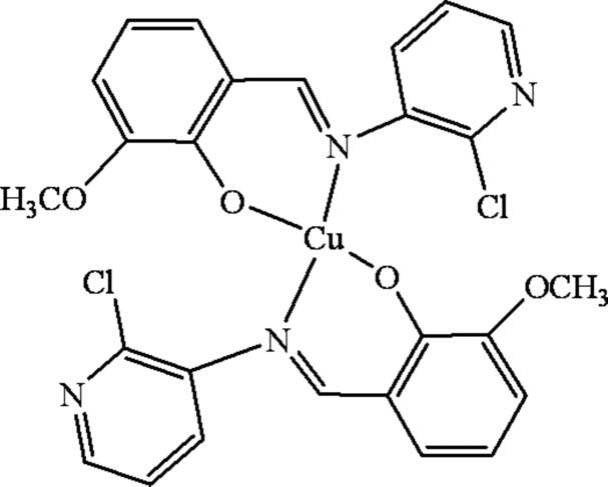


## Experimental

### 

#### Crystal data

[Cu(C_13_H_10_ClN_2_O_2_)_2_]
*M* *_r_* = 586.90
Monoclinic, 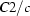

*a* = 21.242 (2) Å
*b* = 7.5743 (9) Å
*c* = 16.141 (2) Å
β = 97.652 (1)°
*V* = 2573.9 (5) Å^3^
*Z* = 4
Mo *K*α radiation
μ = 1.10 mm^−1^
*T* = 298 K
0.18 × 0.16 × 0.11 mm

#### Data collection

Siemens SMART 1000 CCE diffractometer
Absorption correction: multi-scan (*SADABS*; Sheldrick, 1996 ▶) *T*_min_ = 0.827, *T*_max_ = 0.889
6295 measured reflections
2262 independent reflections
1651 reflections with *I* > 2σ(*I*)
*R*_int_ = 0.034

#### Refinement

*R*[*F* ^2^ > 2σ(*F* ^2^)] = 0.035
*wR*(*F* ^2^) = 0.075
*S* = 1.01
2262 reflections
170 parameters
H-atom parameters constrained
Δρ_max_ = 0.24 e Å^−3^
Δρ_min_ = −0.29 e Å^−3^

### 

Data collection: *SMART* (Siemens, 1996 ▶); cell refinement: *SAINT* (Siemens, 1996 ▶); data reduction: *SAINT*; program(s) used to solve structure: *SHELXS97* (Sheldrick, 2008 ▶); program(s) used to refine structure: *SHELXL97* (Sheldrick, 2008 ▶); molecular graphics: *SHELXTL* (Sheldrick, 2008 ▶); software used to prepare material for publication: *SHELXTL*.

## Supplementary Material

Crystal structure: contains datablocks global, I. DOI: 10.1107/S1600536810052414/om2379sup1.cif

Structure factors: contains datablocks I. DOI: 10.1107/S1600536810052414/om2379Isup2.hkl

Additional supplementary materials:  crystallographic information; 3D view; checkCIF report

## Figures and Tables

**Fig. 1.**
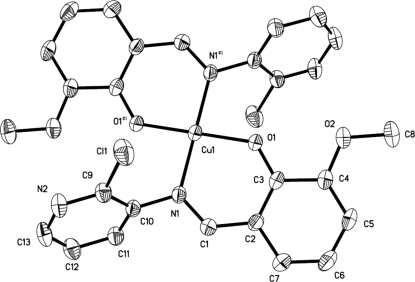
The molecular structure of the title complex with the atom numbering scheme [Symmetry code: #1 = -x + 1,-y + 1,-z]. Displacement ellipsoids for non-hydrogen atoms are drawn at the 30% probability level.

## supplementary crystallographic information

### Comment

The centrosymmetric structure of the title complex is shown in Fig. 1. In the title complex all bond lengths are in normal ranges. The Cu^II^ ion, lying on the inversion centre, is four-coordinated in a *trans*-CuN_2_O_2_ square-planar geometry, with two phenolate O and two imino N atoms from two N,*O*-bidentate Schiff-base ligand (HL) (Dong *et al.*, 2009; Ding *et al.*, 2009). The shortest Cu···Cu distance is 7.5743 (9) Å. However, there are weak intramolecular electrostatic interactions between the Cu and Cl of the ligand, with Cu1··· Cl1 distance of 3.3845 (9) Å.

### Experimental

(*E*)-2-((2-chloropyridin-3-ylimino)methyl)-6-methoxyphenol (HL) was prepared according to previously reported procedure (Dong *et al.*, 2010; Ding *et al.*, 2009). To a warm pale-yellow ethanol solution (4 ml) of 3-methoxysalicylaldehyde (152.2 mg, 1.00 mmol), colorless ethanol solution (4 ml) of 3-amino-2-chloropyridine (128.6 mg, 1.00 mmol) was added dropwise, and the color of the mixture turned orange. The solution was maintained under reflux for 24 h, and a saffron yellow powder product was obtained. It was filtered off, washed with ethanol and ethanol-hexane (1:4, V/V), respectively, and then dried *in vacuo* yielding 245.3 mg powder. Yield, 93.38%. m.p. 397–398 K. Anal. Calcd. for C_13_H_11_ClN_2_O_2_ (%): C, 59.44; H, 4.22; N, 10.66. Found: C, 59.40; H, 4.18; N, 10.71.

A pale-blue ethanol solution (3 ml) of Cu^II^ acetate monohydrate (2.9 mg, 0.015 mmol) was added dropwise to a pale-yellow acetone solution (3 ml) of HL (7.0 mg, 0.027 mmol) at room temperature. The color of the mixing solution turned to yellow immediately, then turned to brown slowly and the filtrate was allowed to stand at room temperature for about three weeks. The solvent was partially evaporated and obtained green single crystals suit for X-ray crystallographic analysis. Anal. Calcd. for [Cu(*L*)_2_] (C_26_H_20_Cl_2_CuN_4_O_4_) (%): C, 53.21; H, 3.43; N, 9.55; Cu, 10.83. Found: C, 53.24; H, 3.46; N, 9.50, Cu, 10.79.

### Refinement

H atoms were placed in calculated positions and non-H atoms were refined anisotropically. H atoms were treated as riding atoms with distances C—H = 0.96 Å (CH_3_) and 0.93 Å (CH). The isotropic displacement parameters for all H atoms were set equal to 1.2 or 1.5 *U*_eq_ of the carrier atom.

### Crystal data

**Table:**

[Cu(C_13_H_10_ClN_2_O_2_)_2_]	*F*(000) = 1196
*M**_r_* = 586.90	*D*_x_ = 1.515 Mg m^−^^3^
Monoclinic, *C*2/*c*	Mo *K*α radiation, λ = 0.71073 Å
Hall symbol: -C 2yc	Cell parameters from 1824 reflections
*a* = 21.242 (2) Å	θ = 2.9–25.2°
*b* = 7.5743 (9) Å	µ = 1.10 mm^−^^1^
*c* = 16.141 (2) Å	*T* = 298 K
β = 97.652 (1)°	Block, brown
*V* = 2573.9 (5) Å^3^	0.18 × 0.16 × 0.11 mm
*Z* = 4	

### Data collection

**Table:**

Bruker SMART 1000 diffractometer	2262 independent reflections
Radiation source: fine-focus sealed tube	1651 reflections with *I* > 2σ(*I*)
graphite	*R*_int_ = 0.034
φ and ω scans	θ_max_ = 25.0°, θ_min_ = 1.9°
Absorption correction: multi-scan (*SADABS*; Sheldrick, 1996)	*h* = −20→25
*T*_min_ = 0.827, *T*_max_ = 0.889	*k* = −8→8
6295 measured reflections	*l* = −19→17

### Refinement

**Table:**

Refinement on *F*^2^	Primary atom site location: structure-invariant direct methods
Least-squares matrix: full	Secondary atom site location: difference Fourier map
*R*[*F*^2^ > 2σ(*F*^2^)] = 0.035	Hydrogen site location: inferred from neighbouring sites
*wR*(*F*^2^) = 0.075	H-atom parameters constrained
*S* = 1.01	*w* = 1/[σ^2^(*F*_o_^2^) + (0.0306*P*)^2^] where *P* = (*F*_o_^2^ + 2*F*_c_^2^)/3
2262 reflections	(Δ/σ)_max_ < 0.001
170 parameters	Δρ_max_ = 0.24 e Å^−^^3^
0 restraints	Δρ_min_ = −0.29 e Å^−^^3^

### Special details

**Table:**

Geometry. All e.s.d.'s (except the e.s.d. in the dihedral angle between two l.s. planes) are estimated using the full covariance matrix. The cell e.s.d.'s are taken into account individually in the estimation of e.s.d.'s in distances, angles and torsion angles; correlations between e.s.d.'s in cell parameters are only used when they are defined by crystal symmetry. An approximate (isotropic) treatment of cell e.s.d.'s is used for estimating e.s.d.'s involving l.s. planes.
Refinement. Refinement of *F*^2^ against ALL reflections. The weighted *R*-factor *wR* and goodness of fit *S* are based on *F*^2^, conventional *R*-factors *R* are based on *F*, with *F* set to zero for negative *F*^2^. The threshold expression of *F*^2^ > σ(*F*^2^) is used only for calculating *R*-factors(gt) *etc*. and is not relevant to the choice of reflections for refinement. *R*-factors based on *F*^2^ are statistically about twice as large as those based on *F*, and *R*- factors based on ALL data will be even larger.

### Fractional atomic coordinates and isotropic or equivalent isotropic displacement parameters (Å^2^)

**Table:**

	*x*	*y*	*z*	*U*_iso_*/*U*_eq_	
Cu1	0.5000	0.5000	0.0000	0.04162 (16)	
Cl1	0.41342 (4)	0.44977 (10)	0.15993 (6)	0.0749 (3)	
N1	0.53184 (10)	0.3389 (3)	0.09230 (13)	0.0415 (5)	
N2	0.40093 (12)	0.1120 (3)	0.17745 (16)	0.0590 (7)	
O1	0.56672 (8)	0.6625 (2)	0.03158 (12)	0.0494 (5)	
O2	0.65231 (10)	0.9115 (3)	0.03223 (14)	0.0662 (6)	
C1	0.58981 (13)	0.3453 (4)	0.13165 (17)	0.0452 (7)	
H1	0.6021	0.2534	0.1685	0.054*	
C2	0.63603 (12)	0.4787 (4)	0.12382 (16)	0.0442 (7)	
C3	0.62132 (12)	0.6322 (4)	0.07655 (16)	0.0426 (7)	
C4	0.67016 (13)	0.7650 (4)	0.07845 (18)	0.0495 (7)	
C5	0.72880 (14)	0.7374 (4)	0.12438 (19)	0.0586 (8)	
H5	0.7603	0.8228	0.1244	0.070*	
C6	0.74167 (15)	0.5833 (4)	0.1708 (2)	0.0642 (9)	
H6	0.7815	0.5668	0.2015	0.077*	
C7	0.69649 (14)	0.4579 (4)	0.17153 (18)	0.0572 (8)	
H7	0.7052	0.3568	0.2037	0.069*	
C8	0.69847 (17)	1.0482 (4)	0.0302 (2)	0.0785 (11)	
H8A	0.7120	1.0891	0.0860	0.118*	
H8B	0.6801	1.1444	−0.0034	0.118*	
H8C	0.7344	1.0028	0.0066	0.118*	
C9	0.43771 (13)	0.2320 (4)	0.14983 (17)	0.0476 (7)	
C10	0.49353 (13)	0.1971 (3)	0.11672 (16)	0.0427 (7)	
C11	0.50962 (14)	0.0223 (4)	0.10883 (17)	0.0516 (7)	
H11	0.5457	−0.0086	0.0853	0.062*	
C12	0.47121 (16)	−0.1066 (4)	0.13646 (19)	0.0637 (9)	
H12	0.4813	−0.2255	0.1321	0.076*	
C13	0.41833 (17)	−0.0569 (4)	0.1702 (2)	0.0647 (9)	
H13	0.3931	−0.1446	0.1891	0.078*	

### Atomic displacement parameters (Å^2^)

**Table:**

	*U*^11^	*U*^22^	*U*^33^	*U*^12^	*U*^13^	*U*^23^
Cu1	0.0330 (3)	0.0411 (3)	0.0496 (3)	−0.0060 (2)	0.0010 (2)	0.0007 (2)
Cl1	0.0740 (6)	0.0506 (5)	0.1082 (7)	−0.0009 (4)	0.0415 (5)	−0.0033 (4)
N1	0.0370 (13)	0.0402 (13)	0.0472 (13)	−0.0072 (10)	0.0046 (11)	−0.0018 (10)
N2	0.0575 (16)	0.0524 (17)	0.0704 (18)	−0.0181 (14)	0.0207 (14)	−0.0051 (14)
O1	0.0380 (11)	0.0423 (11)	0.0642 (12)	−0.0088 (9)	−0.0065 (10)	0.0060 (9)
O2	0.0574 (14)	0.0533 (13)	0.0852 (16)	−0.0221 (11)	−0.0004 (12)	0.0056 (12)
C1	0.0438 (17)	0.0451 (17)	0.0461 (17)	−0.0024 (14)	0.0034 (14)	0.0014 (13)
C2	0.0347 (15)	0.0508 (18)	0.0458 (15)	−0.0041 (14)	0.0009 (12)	−0.0008 (14)
C3	0.0342 (15)	0.0491 (17)	0.0437 (17)	−0.0069 (13)	0.0028 (14)	−0.0073 (14)
C4	0.0444 (18)	0.0483 (19)	0.0557 (19)	−0.0108 (14)	0.0059 (15)	−0.0065 (15)
C5	0.0393 (18)	0.065 (2)	0.070 (2)	−0.0177 (15)	0.0003 (16)	−0.0121 (17)
C6	0.0391 (19)	0.077 (2)	0.072 (2)	−0.0053 (17)	−0.0096 (16)	−0.0098 (19)
C7	0.0467 (19)	0.063 (2)	0.0586 (19)	−0.0025 (15)	−0.0063 (15)	0.0012 (15)
C8	0.082 (3)	0.060 (2)	0.095 (3)	−0.0335 (19)	0.016 (2)	−0.0060 (19)
C9	0.0486 (18)	0.0405 (17)	0.0553 (18)	−0.0050 (14)	0.0125 (15)	−0.0034 (14)
C10	0.0403 (17)	0.0442 (17)	0.0425 (16)	−0.0093 (13)	0.0016 (13)	0.0005 (13)
C11	0.0503 (18)	0.0469 (18)	0.0583 (18)	−0.0059 (15)	0.0096 (14)	−0.0011 (15)
C12	0.073 (2)	0.0439 (19)	0.075 (2)	−0.0104 (17)	0.0156 (19)	−0.0005 (17)
C13	0.075 (2)	0.047 (2)	0.076 (2)	−0.0255 (17)	0.022 (2)	−0.0016 (16)

### Geometric parameters (Å, °)

**Table:**

Cu1—O1^i^	1.8951 (17)	C4—C5	1.378 (4)
Cu1—O1	1.8951 (17)	C5—C6	1.395 (4)
Cu1—N1	1.974 (2)	C5—H5	0.9300
Cu1—N1^i^	1.974 (2)	C6—C7	1.351 (4)
Cl1—C9	1.743 (3)	C6—H6	0.9300
N1—C1	1.309 (3)	C7—H7	0.9300
N1—C10	1.434 (3)	C8—H8A	0.9600
N2—C9	1.314 (3)	C8—H8B	0.9600
N2—C13	1.341 (4)	C8—H8C	0.9600
O1—C3	1.304 (3)	C9—C10	1.389 (4)
O2—C4	1.363 (3)	C10—C11	1.378 (4)
O2—C8	1.429 (3)	C11—C12	1.384 (4)
C1—C2	1.426 (3)	C11—H11	0.9300
C1—H1	0.9300	C12—C13	1.365 (4)
C2—C3	1.403 (4)	C12—H12	0.9300
C2—C7	1.416 (4)	C13—H13	0.9300
C3—C4	1.443 (4)		
			
O1^i^—Cu1—O1	180.00 (14)	C7—C6—C5	120.3 (3)
O1^i^—Cu1—N1	88.30 (8)	C7—C6—H6	119.9
O1—Cu1—N1	91.70 (8)	C5—C6—H6	119.9
O1^i^—Cu1—N1^i^	91.70 (8)	C6—C7—C2	120.8 (3)
O1—Cu1—N1^i^	88.30 (8)	C6—C7—H7	119.6
N1—Cu1—N1^i^	180.00 (16)	C2—C7—H7	119.6
C1—N1—C10	115.2 (2)	O2—C8—H8A	109.5
C1—N1—Cu1	123.28 (18)	O2—C8—H8B	109.5
C10—N1—Cu1	121.36 (17)	H8A—C8—H8B	109.5
C9—N2—C13	116.5 (3)	O2—C8—H8C	109.5
C3—O1—Cu1	127.80 (17)	H8A—C8—H8C	109.5
C4—O2—C8	117.4 (2)	H8B—C8—H8C	109.5
N1—C1—C2	126.7 (3)	N2—C9—C10	125.1 (3)
N1—C1—H1	116.7	N2—C9—Cl1	115.2 (2)
C2—C1—H1	116.7	C10—C9—Cl1	119.6 (2)
C3—C2—C7	120.6 (3)	C11—C10—C9	117.0 (2)
C3—C2—C1	121.9 (2)	C11—C10—N1	122.5 (2)
C7—C2—C1	117.3 (3)	C9—C10—N1	120.5 (2)
O1—C3—C2	124.5 (2)	C10—C11—C12	119.0 (3)
O1—C3—C4	118.2 (3)	C10—C11—H11	120.5
C2—C3—C4	117.3 (2)	C12—C11—H11	120.5
O2—C4—C5	125.7 (3)	C13—C12—C11	119.1 (3)
O2—C4—C3	114.2 (2)	C13—C12—H12	120.5
C5—C4—C3	120.1 (3)	C11—C12—H12	120.5
C4—C5—C6	121.0 (3)	N2—C13—C12	123.3 (3)
C4—C5—H5	119.5	N2—C13—H13	118.3
C6—C5—H5	119.5	C12—C13—H13	118.3
			
O1^i^—Cu1—N1—C1	161.5 (2)	O2—C4—C5—C6	178.8 (3)
O1—Cu1—N1—C1	−18.5 (2)	C3—C4—C5—C6	−1.2 (4)
O1^i^—Cu1—N1—C10	−13.9 (2)	C4—C5—C6—C7	0.0 (5)
O1—Cu1—N1—C10	166.1 (2)	C5—C6—C7—C2	1.5 (5)
N1—Cu1—O1—C3	21.0 (2)	C3—C2—C7—C6	−1.7 (4)
N1^i^—Cu1—O1—C3	−159.0 (2)	C1—C2—C7—C6	−176.5 (3)
C10—N1—C1—C2	−175.3 (2)	C13—N2—C9—C10	−2.2 (5)
Cu1—N1—C1—C2	9.1 (4)	C13—N2—C9—Cl1	179.7 (2)
N1—C1—C2—C3	6.3 (4)	N2—C9—C10—C11	3.2 (4)
N1—C1—C2—C7	−179.0 (3)	Cl1—C9—C10—C11	−178.8 (2)
Cu1—O1—C3—C2	−13.0 (4)	N2—C9—C10—N1	−176.8 (3)
Cu1—O1—C3—C4	167.22 (18)	Cl1—C9—C10—N1	1.2 (4)
C7—C2—C3—O1	−179.3 (2)	C1—N1—C10—C11	−60.3 (3)
C1—C2—C3—O1	−4.8 (4)	Cu1—N1—C10—C11	115.4 (3)
C7—C2—C3—C4	0.4 (4)	C1—N1—C10—C9	119.7 (3)
C1—C2—C3—C4	175.0 (2)	Cu1—N1—C10—C9	−64.6 (3)
C8—O2—C4—C5	0.7 (4)	C9—C10—C11—C12	−2.2 (4)
C8—O2—C4—C3	−179.2 (2)	N1—C10—C11—C12	177.8 (2)
O1—C3—C4—O2	0.7 (4)	C10—C11—C12—C13	0.4 (5)
C2—C3—C4—O2	−179.0 (2)	C9—N2—C13—C12	0.2 (5)
O1—C3—C4—C5	−179.2 (2)	C11—C12—C13—N2	0.6 (5)
C2—C3—C4—C5	1.0 (4)		

Symmetry codes: (i) −*x*+1, −*y*+1, −*z*.
